# Iodine Deficiency in Patients with Hypothyroidism: A Pilot Study

**DOI:** 10.1155/2022/4328548

**Published:** 2022-06-02

**Authors:** Kristin Mariëlla van Veggel, Dina Mehus Ivarson, Jan Maria Martinus Rondeel, Gerritje Sophie Mijnhout

**Affiliations:** ^1^Department of Internal Medicine, Amsterdam UMC-VUmc, De Boelelaan 1117, 1081 HV Amsterdam, Netherlands; ^2^Department of Obstetrics and Gynaecology, Stavanger University Hospital, Gerd-Ragna Bloch Thorsens gate 8, 4011 Stavanger, Norway; ^3^Department of Clinical Chemistry, Isala Clinics, Dokter van Heesweg 2, 8025 AB Zwolle, Netherlands; ^4^Department of Internal Medicine, Isala Clinics, Dokter van Heesweg 2, 8025 AB Zwolle, Netherlands

## Abstract

**Objective:**

Worldwide, 21 countries have insufficient iodine in their diets. Persistent iodine deficiency may result in hypothyroidism. The aim of this study is to determine whether iodine measurements can be used to determine the prevalence of iodine deficiency in patients with (subclinical) hypothyroidism compared to a control group.

**Design:**

A prospective cohort pilot study was performed at the Internal Medicine Outpatient Clinic of Isala, a large teaching hospital in Zwolle, the Netherlands. *Patients*. This study consisted of two groups of 24 adult patients each: a group of consecutive patients presenting with overt or subclinical hypothyroidism and a control group of euthyroid patients with type 1 diabetes mellitus. *Measurements*. All patients collected 24-hour urine. Iodine status was determined using urinary iodine concentration (UIC), urinary iodine excretion (UIE), and iodine creatinine ratio (I : Cr). Iodine deficiency was defined as an iodine concentration <100 *µ*g/L for UIC, iodine level <125 *µ*g for UIE, and <0.13 *µ*mol/mmol for I : Cr.

**Results:**

According to UIE and UIC measurements, 54.2% of hypothyroid patients were iodine-deficient compared to 41.7–45.8% in the control group. According to the I : Cr measurement 91.7% of hypothyroid patients were iodine-deficient compared to 87.5% in the control group. No significant difference was seen between the two groups. No correlation was found between thyroid-stimulating hormone (TSH) level and iodine deficiency.

**Conclusions:**

Iodine deficiency is prevalent in both hypothyroid patients and euthyroid patients. Because there is no significant difference between the groups, a single 24-hour urine or spot urine sample to determine UIC, UIE, and I : Cr, seems not suitable to determine iodine status in an individual participant.

## 1. Introduction

Iodine is an essential component of the hormones thyroxine (T4) and triiodothyronine (T3), which are produced by the thyroid gland [1]. These hormones regulate metabolism and promote growth, development, and maturation of all organs, especially the brain [2]. Persistent iodine deficiency and therefore low thyroid hormone synthesis may result in hypothyroidism. Iodine deficiency may also cause so called iodine deficiency disorders, which include an increased risk of early miscarriage and preterm delivery in pregnant women, low birth weight, and increased risk of impaired mental function as well as goiter development [3].

Iodine deficiency is still prevalent in all regions worldwide, and it affects populations at all stages of economic development. In 2020, worldwide, 21 countries have insufficient iodine in their diets. The iodine intake is lowest in Madagascar, but countries in Europe (Germany and Finland) also have a deficient iodine intake [4].

The native iodine content of most foods and drinks is low. The most important natural sources of iodine are seafood, dairy products, and vegetables grown from iodine-rich soil. In many countries, the use of iodized salt provides additional iodine [5].

There are several ways to determine iodine status in humans. One way is to calculate median urinary iodine concentration (UIC) from spot samples, a method that is used by the World Health Organization (WHO) for population screening purposes in their report on iodine deficiency [6]. A median UIC >100 *µ*g/L is considered sufficient. Deficiency is classified as mild (UIC 50–99 *µ*g/L), moderate (UIC 20–49 *μ*g/l) or severe (UIC <20 *μ*g/l). In order to ensure a sufficient iodine status, the WHO recommends a dietary intake of iodine contains 150 *µ*g for adults and 250 *µ*g for pregnant or lactating women [6]. Another option to determine iodine status is to measure urinary iodine excretion (UIE) over 24 hours by performing a 24-hour urine collection and determining its total iodine content. The advantage of this method is that all excreted iodine is collected, and will therefore be minimally affected by diurnal variation. Therefore, 24-hour UIE for evaluating iodine status is in some studies chosen over other methods [7, 8]. 90% of ingested iodine is excreted within 24 hours [9]. A low amount of iodine in the 24-hour UIE could therefore indicate a low intake and thus deficiency, while a high amount of iodine in the urine could reflect a higher intake. A disadvantage of this method is that it is based on recent iodine intake (days) and does not reflect long-term intake [9]. Also, this method does not correct for variation in daily intake [10]. Another option is a spot urine sample where the urine iodine concentration is scaled to urine creatinine concentration (I : Cr). This test is easy and practical to perform and has proved to give a result closer to the UIE compared to what can be achieved with the UIC [7].

Guidelines regarding hypothyroidism by the American Thyroid Association and the European Thyroid Association do not mention the evaluation of iodine status [11, 12]. Therefore, it is possible that cases of iodine deficiency-induced hypothyroidism remain undiagnosed. If the treating physician would be aware of this underlying cause, these patients could be treated with iodine supplementation instead of lifelong therapy with levothyroxine.

The aim of this pilot study is to investigate whether iodine measurements can be used to determine the prevalence of iodine deficiency in patients with (subclinical) hypothyroidism compared to a control group.

## 2. Materials and Methods

### 2.1. Study Design and Settings

A prospective cohort pilot study was performed at the Internal Medicine Outpatient Clinic of Isala. Isala Clinics is a large teaching hospital in Zwolle, the Netherlands.

### 2.2. Patients

This study comprised two groups. Group one consisted of adult patients presenting with overt or subclinical hypothyroidism ((S)H). Overt hypothyroidism was defined as thyroid-stimulating hormone (TSH) > 4.0 mU/L and free thyroxine (FT4) < 10.0 pmol/L, while subclinical hypothyroidism was defined as TSH >4.0 mU/L and FT4 within the normal reference range of 10.0–24.0 pmol/L. Group two (the control group) consisted of adult patients with type 1 diabetes mellitus (T1DM) and a normal thyroid function. The reason we choose patients with T1DM is that in this patient group, the thyroid function is checked regularly and thus was known to us. In addition, most T1DM patients are familiar with 24-hour urine collection. The patients in the control group were matched individually on gender and age (±two years) with the patients in group one.

We excluded patients on medication or supplements containing iodine or affecting the thyroid function in other ways (e.g., levothyroxine, lithium, amiodarone, and iodine-containing multivitamins). We also excluded pregnant patients (due to the physiologically increased need for iodine) and patients with renal impairment.

We included patients in groups one and two between the 6th of November 2017 and the 30th of October 2018.

### 2.3. Methods

In all patients of group one, TSH and FT4 were determined. Anti-thyroid peroxidase (anti-TPO) antibodies were determined if this has not already been conducted by the general practitioner, by whom the patient was referred. The medical records of the patients of both groups were evaluated for age, sex, BMI, smoking, alcohol use, cardiovascular disease, and goiter (determined by physical examination and/or ultrasound).

All patients performed a 24-hour urine collection. The total volume and creatinine concentration of the urine sample was measured at the department of clinical chemistry in Isala Clinics. A 10 ml extraction from the sample was sent to the laboratory of the Academic Medical Centre in Amsterdam for analysis of UIC. With these values, we calculated UIE and I : Cr.

Iodine deficiency was defined as an iodine level <100 *µ*g/L for UIC, an iodine level <125 *µ*g for UIE, and <0.13 *µ*mol/mmol for I : Cr [13]. Adequacy of collection of 24 h urine was assured by determining volume and creatinine concentration.

TSH and FT4 were measured on a Roche Cobas 8000 platform. Both assays are solid-phase electrochemical luminescent immunoassays. Intra- and interassay coefficients of variance amount to 2%. Creatinine is measured by an enzymatic method on a Cobas platform (Roche). The clinical chemistry departments are accredited under NEN-EN-ISO 15189:2012. Urinary iodine was measured spectrophotometrically according to the Sandell–Kolthoff method. Potassium iodide was used as standard (Sigma 1.05043, ≥99.5%). The detection limit of the assay is 39 *µ*g/L at which precision is 15%. In each run, internal urine controls were run at two levels. Proficiency schedule samples (CDC Equip) were run three times a year.

### 2.4. Data Analysis and Statistics

All data were gathered from the institution's electronic patient file system.

IBM SPSS version 26 was used for data analysis.

Descriptive statistics were used to get an overview of the baseline characteristics of the two groups. Since none of the variables showed a Gaussian distribution, all performed tests were nonparametric. The data shown are median (interquartile range). Fisher exact test and Mann–Whitney *U* test have been used.

The relation between iodine deficiency and (S)H was analyzed as categorical data with binomial distribution using Fisher exact test. Fisher exact test was chosen given the small sample size and because normal distribution was not assumed.

In order to investigate whether there was a correlation between the iodine content of the urine samples and TSH level, a Spearman's rank correlation coefficient analysis was performed.

This analysis was chosen given the assumptions of non-normal data distribution.

A *P* value of less than 0.05 was regarded as statistically significant.

The prevalence of iodine deficiency among patients with hypothyroidism was not exactly known. A reliable prevalence for the general population was also lacking. It was therefore not possible to perform a power analysis to estimate the sample size necessary to find a significant difference. We accepted this given that this study is a pilot study meant to prepare for a larger-scale study.

### 2.5. Medical Ethical Committee

The Medical Ethical Committee of Isala Clinics granted permission to perform the study, reference number 171017.

## 3. Results

### 3.1. Patient Inclusion

All new patients who presented at the internal medicine outpatient clinic of Isala with (S)H in the given inclusion period and met the inclusion criteria were evaluated to participate in the study. A total of 43 patients met the inclusion criteria, and of these patients, 28 patients were included for analysis. After analysis, four patients were excluded because there was doubt about the adequacy of the collection of 24-hour urine because of a deviating 24-hour volume. A total of 24 patients were included in this study.

**Figure 1:**
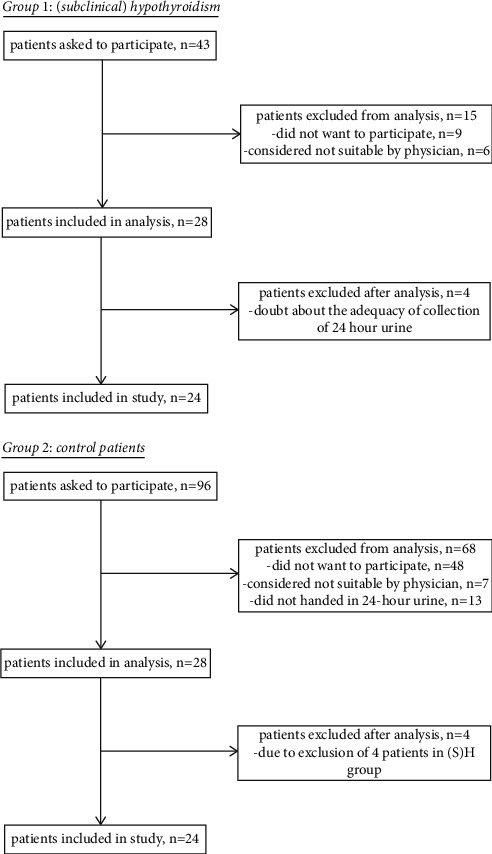
Inclusion procedure.

Also, all patients who met the inclusion criteria for the control group in the given inclusion period were evaluated to participate in this study. A total of 96 patients met the inclusion criteria of which 28 patients were included and could be matched with the patients in group one. At the end, we reduced this group to 24 patients, because of the fact that four patients from the (S)H group were excluded due to doubt about the adequacy of collection of 24-hour urine (see Figure 1).

### 3.2. Baseline Characteristics

The baseline characteristics are presented in Table 1.

In the (S)H group, 87.5% had subclinical hypothyroidism and 12.5% had overt hypothyroidism.

### 3.3. Iodine Deficiency

As displayed in Table 2, the UIE was not significantly different between group one and group two (cutoff value < 125 *µ*g). In the (S)H group, 54.2% of the patients had iodine deficiency, compared to 41.7% of the control group (no significant difference).

Using different cutoff points for iodine deficiency (<50 *µ*g, <75 *µ*g, and <100 *µ*g) also did not show a significant difference between the (S)H group and the control group. No patients in either group had a UIE <25 *µ*g.

The UIC was also not significantly different between both groups. Using this method, 54.2% of the (S)H group had iodine deficiency compared to 45.8% in the control group.

Using a cutoff value of <0.13 in the I : CR, 91.7% in the (S)H group had iodine deficiency compared to 87.5% in the control group (no significant difference).

No significant correlation was found between the TSH level and UIE, TSH level and UIC, and TSH level and I : Cr (Table 3).

## 4. Discussion/Conclusion

Iodine deficiency is one of the main risks to develop hypothyroidism. Interestingly, this study showed that iodine deficiency was very common in both groups, regardless of the method used to evaluate iodine status. A possible explanation for this could be the general trend in our society to eat less salt, less bread, and more organic foods, which all contribute to a lower iodine intake. This has been confirmed by a previous study on iodine intake in the Netherlands: most of the Dutch population had an inadequate habitual iodine intake (85% of females and 67% of males) [14]. Another explanation could be that the iodine concentration in fortified salt was reduced in 2008 [14]. Therefore, a larger part of the population would be at risk for having iodine deficiency, not only patients with (S)H and T1DM.

In all three methods to evaluate iodine status, we did not find a significant difference in the prevalence of iodine deficiency between the two groups. There was also no correlation between TSH level and UIE, TSH level and UIC, and TSH level and I : Cr. Although it would be likely to believe that iodine deficiency would be more common in the (S)H group, it is not possible to draw final conclusions based on our results because of the small size of this study. Theoretically, it is still probable that the prevalence of iodine deficiency among patients with (S)H is higher than that in the euthyroid population as hypothyroidism is part of the symptomatology of iodine deficiency [3]. This hypothesis is supported by several previous studies [15–18].

Despite the fact that the overall prevalence of iodine deficiency in the control group in this study is high, it is questionable whether this is representative of the rest of the euthyroid population. Our control group consisted of patients with T1DM. There are no documented effects of T1DM on iodine metabolism. Therefore, it seems unlikely that T1DM may influence iodine intake or excretion.

Even though iodine deficiency is an important global issue, there is still discussion about the best method of evaluating iodine status as well as the significance of an individual outcome of such a test. The first method used in this study is 24-hour UIE. The urine iodine content is difficult to interpret because of the quick iodine excretion from the blood into the urine. As 90% of iodine is excreted within 24 hours, urine iodine content is an accurate way to measure recent iodine intake; however, it does not correct for the large variation in daily intake. It also does not describe long-term trends. With this uncertainty regarding the interpretation of iodine measurements, repeated 24-hour urine samples would offer more reliability. Determination of iodine deficiency by UIC shows almost the same results as the determination by UIE. Therefore, the determination of iodine deficiency using a spot urine sample, instead of 24-hour urine, seems sufficient. However, the cutoff value for iodine deficiency measured by UIC we used and which was suggested by the WHO (<100 *µ*g/L), might be too high. Zimmerman and Andersson state that a UIC <100 *µ*g/L is applicable in children, but not in adults. A cutoff value of 60–70 *µ*g/L for adults is suggested [10]. Using a lower cutoff value would result in fewer patients with iodine deficiency. Determination of iodine deficiency by I : Cr also showed more iodine deficiency in the (S)H group compared to the control group; however, this difference was not significant. It is remarkable that the percentage of patients with iodine deficiency in both the (S)H group and the control group are much higher using the I : Cr method than the UIE and UIC to determine iodine deficiency. In contrast to what is discussed in other studies [7], this study does not show that the I : Cr method gives results closer to the UIE compared to what can be achieved with the UIC. The reason for this may be that the cutoff value for iodine deficiency we used is too high since the percentages in both UIC and UIE correlated well.

Since one would suspect a lower prevalence of iodine deficiency in the control group compared to the (S)H group, it is also possible that a single 24-hour urine or spot urine sample to determine UIC, UIE, and I : Cr is not suitable to determine iodine status in an individual participant (as it is for a population). At the moment, there are no standardized alternatives to determine iodine deficiency in the individual patient.

Serum iodine might be used to evaluate iodine deficiency, but no reference ranges are available for this purpose. It is also questionable whether serum levels are more representative of long-term iodine intake than urine iodine given the rapid renal iodine filtration and excretion. Jin et al. found that serum iodine had a strong nonlinear correlation with urinary iodine in adults [19]. They also found that low levels as well as very high levels of serum iodine affect thyroid health (excessive amounts of serum iodine inhibit the synthesis of thyroid hormones) [19]. Iodine measurement in hair could possibly provide a long-term picture of iodine status over time [20].

To determine population iodine status, the measurement of thyroglobulin is increasingly being used [21]. Mean thyroglobulin concentrations are typically elevated in regions of both iodine deficiency and also excess [22]. Again, the value of thyroglobulin measurement in individual patients to assess iodine status has not been determined.

Based on our study it seems likely that iodine deficiency is equally present among hypothyroid patients as well as euthyroid patients. Despite the small sample size, the percentage of patients with iodine deficiency is surprising. Further research is indicated to examine whether these results can be reproduced in a large-scale study. If our findings are confirmed, we suggest, especially in patients with unexplained hypothyroidism, to check the iodine status. Future research should also include treatment with iodine supplements in the patients with iodine deficiency and hypothyroidism and thereafter follow-up of the thyroid function.

## Figures and Tables

**Table 1:** Baseline characteristics.

		(S)H	Control	*p* value
Diagnosis	Subclinical hypothyroidism	87.5%	0%	NR
	Overt hypothyroidism	12.5%	0%	
	T1DM	0%	100%	

Age (years)	Median	42.5	42.5	0.967^2^
	Min–Max	22–78	23–76	
	IQR	27.5–59.3	27.5–58.5	

Sex	Female (%)	79.2%	79.2%	1.000^1^
	Male (%)	20.8%	20.8%	

BMI (kg/m^2^)	Median	25.4	24.5	0.647^2^
	Min-Max	18–43	19–39	
	IQR	22–29	22–29	

TSH (mU/L)	Median	8.8	2.1	<0.001^2^
	Min–Max	4.1–66	0.5–4.2	
	IQR	6–15.3	1.6–2.5	

Smoking	Percentage	12.5%	4.2%	0.609^1^
Alcohol	Percentage	45.5%	54.5%	0.763^1^
Cardiovascular disease	Yes (%)	8.3%	8.3%	1.000^1^
Goiter^a^	Yes (%)	12.5%		NR
Anti-TPO	Positive (%)	59.1%		NR

^a^By physical examination, ^1^ Fisher exact test, ^2^ Mann–Whitney U test; (S)H, overt or subclinical hypothyroidism; T1DM, type 1 diabetes mellitus; IQR, interquartile range; BMI, body mass index; TSH, thyroid-stimulating hormone; NR, not reported.

**Table 2:** Prevalence of iodine deficiency.

		(S)H	Control	*p* value
Urinary iodine excretion (*µ*g)	Median	123.2	132.3	0.391^2^
	Min–Max	30.8–725.0	29.3–368.9	
	IQR	93.8–173.4	103.2–180.5	

Iodine deficiency^a^	Yes (%)	54.2%	41.7%	0.564^1^
Urinary iodine	Median	71.5	81.5	0.568^2^
Concentration (*µ*g/L)	Min–Max	38–500	43–188	
	IQR	44–133.5	55.8–112.8	

Iodine deficiency^b^	Yes (%)	54.2%	45.8%	0.773^1^
Iodine creatinine ratio (*µ*mol/mmol)	Median	0.09	0.08	0.640^2^
	Min-Max	0.02–0.56	0.02–0.21	
	IQR	0.06–0.11	0.07–0.12	

Iodine deficiency^c^	Yes (%)	91.7%	87.5%	1.000^1^

^a^Iodine deficiency was defined as an iodine level <125 *µ*g, ^b^Iodine deficiency was defined as an iodine concentration <100 *µ*g/L, ^c^Iodine deficiency was defined as an iodine creatinine ratio <0.13, ^1^Fisher exact test, ^2^Mann–Whitney U test; (S)H, overt or subclinical hypothyroidism.

**Table 3:** Correlation between TSH value and urine iodine excretion.

	Combined correlation coefficient	*p* value	(S)H group correlation coefficient	*p* value	T1DM correlation coefficient	*p* value
Urinary iodine excretion (*µ*g)	−0.127	0.396^3^	0.054	0.801^3^	−0.302	0.161^3^
Urinary iodine concentration (*µ*g/L)	−0.102	0.543^3^	0.021	0.928^3^	−0.041	0.873^3^

Iodine creatinine ratio (*µ*mol/mmol)	−0.148	0.316^3^	−0.064	0.767^3^	−0.383	0.065^3^

TSH, thyroid-stimulating hormone; (S)H, overt or subclinical hypothyroidism; T1DM, type 1 diabetes mellitus; ^3^Spearman's rank correlation coefficient analysis. To convert iodine *µ*g to *µ*mol, a conversion factor of 0.0079 was used.

## Data Availability

The data used to support the findings of this study are available on request from the authors.
